# Quantitative Distribution Profile of Cadmium and Lead in Different Organs of Rats and Mitigation of their Accumulation Through Probiotic Treatment

**DOI:** 10.1080/29933935.2024.2313299

**Published:** 2024-05-02

**Authors:** Omprakash Omprakash, Rohit Kumar, Prashant Singh, Poonam Devi, Anuj Malik, Nitin Mahal

**Affiliations:** a ICAR-National Dairy Research Institute, Animal Biochemistry Division, Karnal, India; b College of Public Health and Human Sciences, Oregon State University, Corvallis, OR, USA

**Keywords:** Cadmium, lead, Wistar rat, antioxidant enzymes, probiotics

## Abstract

Cadmium (Cd) and lead (Pb) are toxic heavy metals that can have severe adverse effect on human health and environment. Pb contamination remains a significant concern due to its persistence in the environment, while Cd primarily enters the environment through mining, contaminating water and soil. These metals have a propensity to accumulate within various organs by displacing the essential divalent cation Ca2+, which plays a crucial role in mammalian physiology. However, the pattern of accumulation is not uniform across different organs and can vary due to distinct processing mechanisms and affinities exhibited by heavy metals toward various organ systems. This present study aims to comprehensively assess the quantitative distribution of cadmium and lead within the organs of Wistar rats, who were administered 50 mg and 100 mg per kilogram of body weight, respectively, of Cd and Pb. Furthermore, the study employs a therapeutic and interventional approach to mitigate heavy metal toxicity by introducing two probiotic strains: *Lactobacillus fermentum* NCDC-400 and *Lactobacillus rhamnosus* NCDC-610. The group of Wistar rats receiving the probiotic treatment demonstrated a noteworthy reduction in the accumulation of Cd and Pb, accompanied by a significant improvement in antioxidant enzyme activities and histological features of liver and kidney.

## Introduction

Heavy industrialization and automation have changed the world during the last century, but human exposure to heavy metals has massively increased health issues such as heavy metal poisoning. Symptoms associated with heavy metal toxicity generally vary with accumulated metals. Some metals, such as iron, zinc, and manganese, are indispensable to human regulatory body function, as they act as enzyme cofactors in very small concentrations. However, few metals, such as cadmium, lead, and arsenic, accumulate in the body in a significant amount, resulting in metal toxicity that may lead to serious organ damage. Among these, exposure to lead and cadmium is widespread due to persistence in our environment, and the low socioeconomic status of the human population increases the risk of heavy metal exposure, resulting in diseases associated with its toxicity.^1^ These heavy metals are reported to adversely affect various body organs, leading to several health-related risks and potential long-term complications.

Pb-induced toxicity is reported to have a multitude of effects on proper cognitive functioning and can cause learning disabilities and alterations in behavior.^2^ Due to its ability to substitute Ca2+, it can easily cross the blood‒brain barrier and can accumulate in the brain and exert neurotoxicity.^3^ It is also reported to disrupt the function of GABAergic, cholinergic and dopaminergic systems by interfering with the release of neurotransmitters. Pb impedes nervous system development from the prenatal period to childhood by interfering with synapse formation and the premature differentiation of glial cells. The severe form of Pb-induced neurotoxicity can cause lead encephalopathy accompanied by symptoms such as headache, mental dullness, attention difficulty, memory loss, tremor and hallucination.^4,5^ Similar to Pb, Cd has also been reported to cross the blood‒brain barrier due to its ability to substitute Ca2+ and thus can elicit neurotoxicity.^6^ The toxicity is exerted by Cd-induced inflammation, oxidative stress, and neuronal apoptosis.^7–9^

Cadmium mostly accumulates in the liver, kidney and testes and has a very low clearing rate from biological systems.^10^ Both Pb- and Cd-induced nephropathy is marked by Fanconi syndrome, which includes polyuria, proteinuria, and glucosuria. The tubular dysfunction associated with Fanconi syndrome is also accompanied by degenerative changes in the tubular epithelium and nuclear inclusion bodies containing Pb. Studies have shown that even low to moderate levels of exposure to Pb and Cd can result in nephrotoxicity and the occurrence of chronic kidney disease (CKD).^11^

Low doses of lead can significantly reduce sperm count as well as motility.^12^ The sperm count, motility, and general morphology were affected when the blood level of lead exceeded 40 μg/dL.^13^ High doses of lead cause a significant increase in the percentage of epididymal abnormal sperm with a significant reduction in viable and motile sperm by targeting testicular spermatogenesis.^12^ In rodents, high doses of cadmium can result in pathological changes such as damage to the testis (germ cell depletion and necrosis), interstitial tissue, and blood‒testis barrier.^14,15^ In humans, Cd exposure significantly affects male reproductive organs and results in impaired spermatogenesis and semen quality. It also compromises the female reproductive system by affecting the menstrual cycle.^16^

The gut microbiota is the first line of immune defense against the toxic effect of heavy metals. The gut microbiota alters the uptake and metabolism of heavy metals by influencing physical barriers to heavy metal absorption and by varying the pH, oxidative status, and even concentrations of detoxification enzymes involved in heavy metal metabolism. This evidence represents consistent bidirectional crosstalk between heavy metals and the gut microbiome. Thus, enhancing gut microbiota diversity may be a proven possible effective and affordable approach for combating toxic metal exposure.^17^ The heavy metals cadmium and lead deposition and their toxicity have been reported in various organs. The present study focuses on the quantitative accumulation pattern of these metals in different organ systems of rats. This study will provide insight into the organs preferred by heavy metals for their accumulation as well as how to remedy their accumulation and toxicity through probiotic intervention.

## Material and methods

### Bacterial strains

A total of two *lactobacilli* strains (*Lactobacillus Fermentum*-NCDC-400 and *Lactobacillus rhamnosus*-NCDC-610) were procured from the National Collection of Dairy Cultures (NCDC), Dairy Microbiology Division, ICAR – National Dairy Research Institute, Karnal − 132001, Haryana, India. These strains were cultured in de Man Rogosa and Sharpe (MRS) broth at 37°C for 18 h. All lactic cultures were subcultured twice before the experiment.

### Preparation of standard and metal stock solutions

Calibration standard solutions of varying concentrations (50, 40, 30, 20, 10 & 1 mg. L-1) was prepared by diluting a Cd and Pb stock solution of 1000 mg/L pure standard solution for atomic absorption spectroscopy (AAS, Sigma) in double-distilled water. The stock solution of Cd and Pb (1000 mg. L-1) for the bioadsorption assay was prepared by dissolving cadmium chloride and lead(II) acetate trihydrate [Pb(C2H_3_O_2_)_2_]; PubChem ID 329,754,133) in 50 mL of 2% nitric acid and then with double distilled water. The desired working solution was prepared by diluting the stock solution for each experiment. The standards were run every time the samples were analyzed with an atomic absorption spectrophotometer.

### Animal experiments

Adult male Wister rats used in the experiments were obtained from the small animal house of the National Dairy Research Institute, Karnal, Haryana (India). Rats were selected strictly by weight (between 100 and 120 g) and age (approximately 8 weeks old). The animals used in the study were kept in stainless steel cages in a temperature- and humidity-controlled room that was able to maintain a 12 h light/dark cycle. The rats were fed standard commercial mouse food, and water was given ad libitum.

#### Protective effects of the selected strain against cadmium and lead exposure

**Figure 1.**
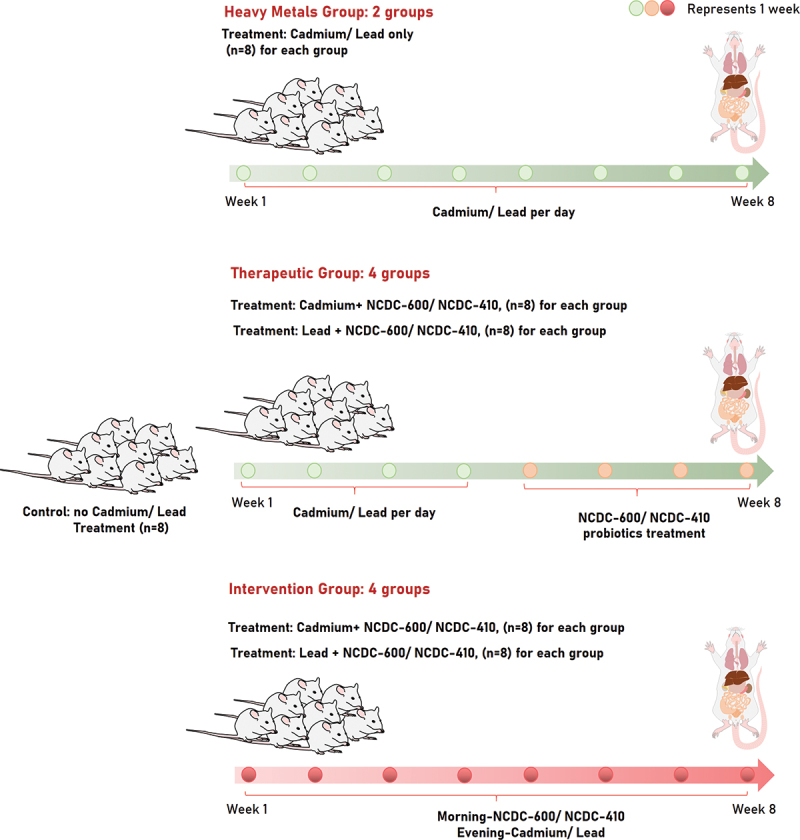
Animal groups consisted of seven Wistar rat groups: (i) a control group receiving no heavy metal treatment, (ii) 2 heavy metal groups receiving Pb and Cd, and (iii) 2 therapeutic groups receiving Pb and Cd, each group administered the NCDC-400 and NCDC-610 probiotic strains four weeks after Pb and Cd treatment (iv) there were 2 intervention groups for Pb and Cd, and each group received the probiotic strains NCDC-400 and NCDC-610 on the same day of Pb and Cd treatment. Each group consisted of eight animals.

Rats were randomly divided into two major groups. Intervention groups were used to investigate the effect of coadministration of lactobacilli and Cd and Pb, while therapy groups were used to study the therapeutic function of lactobacilli after Cd and Pb toxicity was established in rats. Each of these two major groups was divided into six subgroups, consisting of one control group, one heavy metal group, and four heavy metal groups (Pb and Cd receiving two different probiotic strains). The animal grouping and experimental design is illustrated in Figure 1. For both the intervention and therapy groups, the experimental period was 8 weeks. Rats received two lactobacilli strains (NCDC-400 & NCDC-610) after they were exposed to Cd and Pb. The toxic doses of Cd and Pb for rats were designated as 50 mg/kg rat weight and 100 mg/kg rat weight once daily. Rats in both the intervention and therapy groups were given 0.2 mL skim milk containing 2 × 10^10^ CFU/mL lactobacilli by gavage once daily.

During the experimental period, each rat was moved into a clean cage and emptied every week for one hour for the collection of fecal samples. No animal died during the experimental period. At the end of the experiment, the rats were sacrificed under light ether anesthesia, and blood samples were collected in heparinized tubes to obtain plasma. The liver, kidneys, heart, lungs, spleen, testes and brains were excised and washed with saline solution. The tissue samples were then collected in metal-free Eppendorf tubes and stored at −80°C for biochemical assays and estimation of Cd and Pb concentrations. The levels of Cd and Pb in different organ samples were analyzed by flame atomic absorption spectrophotometry (Shimadzu AA-7000). To avoid the contamination of any other metal, feces and tissues were collected in clean metal-free low retention polypropylene Eppendorf tubes. Before the estimation of metal levels, tissues and feces were transferred to digestion vessels and digested in concentrated HNO_3_ using the Microwave Digestion System. The digestion vessels had TFM-wetted surfaces and were washed with ultrapure water thoroughly before use.

### Determination of Cd and Pb in feces and tissue

The method of Palma et al.. (2015) with some modification was used.^18^ To perform the one-step digestion procedures, approximately 250 mg of sample (feces and tissue) was placed in glass tubes. The tissue and fecal samples were then treated with 5 mL of digestion solution (a mixture of nitric and perchloric acid at a ratio of 2:1, 3:1, or 4:1 v/v). The tubes were subsequently heated to 200°C until the solution became translucent, and the emission of brownish smoke ceased, indicating the thorough digestion of the organic matter. The tubes were allowed to cool at room temperature. The digested samples were quantitatively transferred to 50 mL volumetric flasks. The transfer was accomplished using ash-free quantitative filter paper (Whatman No. 41, Whatman International Ltd, Springfield, Kent, and England). The volume of the solutions was made up to 50 mL using deionized water. Aliquots of the solutions were transferred to polyethylene flasks and kept at 4°C. The Cd and Pb concentrations in the treated samples were determined using a flame atomic absorption spectrophotometer (FAAS, Shimadzu AA-7000).

The Cd and Pb present in tissue was determined by the following formula:

$$\mathrm{Retained}\left(\%\right)=100\%\times \left[\left(C0-C1\right)/C0\right]$$

where C_0_ and C_1_ are the initial Cd and Pb concentrations and the residual Cd and Pb concentrations accumulated in the tissue and feces, respectively.

### Measurement of oxidative stress using biochemical markers

#### Antioxidant enzyme estimation

For the measurement of oxidative stress, a 1 g tissue (testis and liver) sample was added to 9 mL PBS solution, and the mixture was homogenized using a homogenizer and then centrifuged at 2500×g for 10 min at 4°C. The supernatant was collected to measure biochemical markers. The total protein was determined using the Lowry method, and the absorbance was measured at 660 nm using a UV/VIS spectrophotometer (Shanghai Metash Instruments Co., Ltd., Shanghai, China). The enzyme activities of SOD, CAT and GSH in the liver and kidney were measured according to catalase and superoxide dismutase (SOD).^19^ The results are expressed as U/mg protein. U was defined as the amount of SOD needed to inhibit pyrogallol by 50% for SOD activity. For the analysis of CAT activity, the reaction mixtures consisted of 2.9 mL of 30 mM hydrogen peroxide, which was preincubated at 25°C for 5 min. The reaction was initiated by the addition of 100 μL of supernatant. One U of CAT activity was defined as a change of 0.01 in absorbance/min at 240 nm using hydrogen peroxide as the substrate. The enzyme activity was expressed as U/mg protein. GPx was assayed utilizing excess glutathione reductase, which couples the rate of oxidation of NADPH to the reaction of peroxidase with H_2_O_2_ and glutathione (reduced). The enzyme activity was calculated using an extinction coefficient of 6.22 mM-1 cm-1, where the unit enzyme activity is one mmol of NADPH oxidized per min or units/mg/min.

#### Malondialdehyde estimation

Lipid peroxidation in tissues and plasma was estimated by the method described by Kaushal and Kansal (2012).^20^ In this method, malondialdehyde and other thiobarbituric acid reactive substances (TBARS) were measured by their reactivity with thiobarbituric acid (TBA) in acidic conditions to generate a pink-colored chromophore, which was measured at 535 nm. The tissue homogenates were prepared in phosphate buffer (50 mM, pH 7.4). To one mL of tissue homogenate or plasma, 2 mL of TCA-TBA-HCl (5% TCA in distilled water + 0.375% TBA in hot distilled water + 0.25 N HCl) reagent was added and mixed thoroughly. The mixture was kept in a boiling water bath for 15 min. After cooling, the tubes were centrifuged at 1000 × g for 10 min, and the color developed in the supernatant was measured in a spectrophotometer at 535 nm against the reagent blank. A series of standard solutions in the range of 8–40 nmol were treated in a similar manner. The standards were prepared by overnight digestion with different concentrations of 1,1,3,3-tetraethoxypropane in the presence of 0.2 N HCl. Values were expressed as nmol/mg protein.

### Histopathology of the liver and kidney of the therapeutic and intervention groups

After the study period, the experimental animals were sacrificed, and their liver and kidney tissues were harvested, weighed and processed for histological staining. During tissue processing, 10% neutral buffered formalin was used to fix the tissue, alcohol was used as a dehydrating agent, and xylene was used as a clearing agent. Processed liver and kidney tissues of experimental animals were embedded in molten paraffin wax and allowed to solidify to form tissue blocks. The liver and kidney tissue blocks were sectioned at 5 μm thickness and stained with hematoxylin and eosin stains for subsequent histomorphological study. Histological assessments were performed under a light microscope (Olympus BX43, Tokyo, Japan).

### Statistical analyses

Effect of *L. Fermentum* (NCDC-400) *and L. rhamnosus* (NCDC-610) strains on the bioaccumulation of Cd and Pb in different organ systems and feces of Wistar rats were analyzed by two way analysis of variance (ANOVA) followed by Tukey’s multiple comparison test. The antioxidant enzyme activity and MDA estimation in liver and kidney tissue data was analyzed by one way ANOVA followed by Tukey’s multiple comparison test. The data’s statistical significance was assessed with a confidence interval of 95% and a significance level of 5%. All data were represented as mean±SEM.

A Python script in Jupyter notebook was employed to create a heatmap illustrating the accumulation profile of Cd and Pb in different organ systems of the Wistar rat group. The Cd and Pb values, determined by atomic absorption spectroscopy for various organ systems, were log-transformed and utilized for heatmap generation. The Viridis color palette was chosen for the visualization of the heatmap.

## Results

### Determination of cadmium and lead in tissues and feces

#### Cadmium

**Figure 2.**
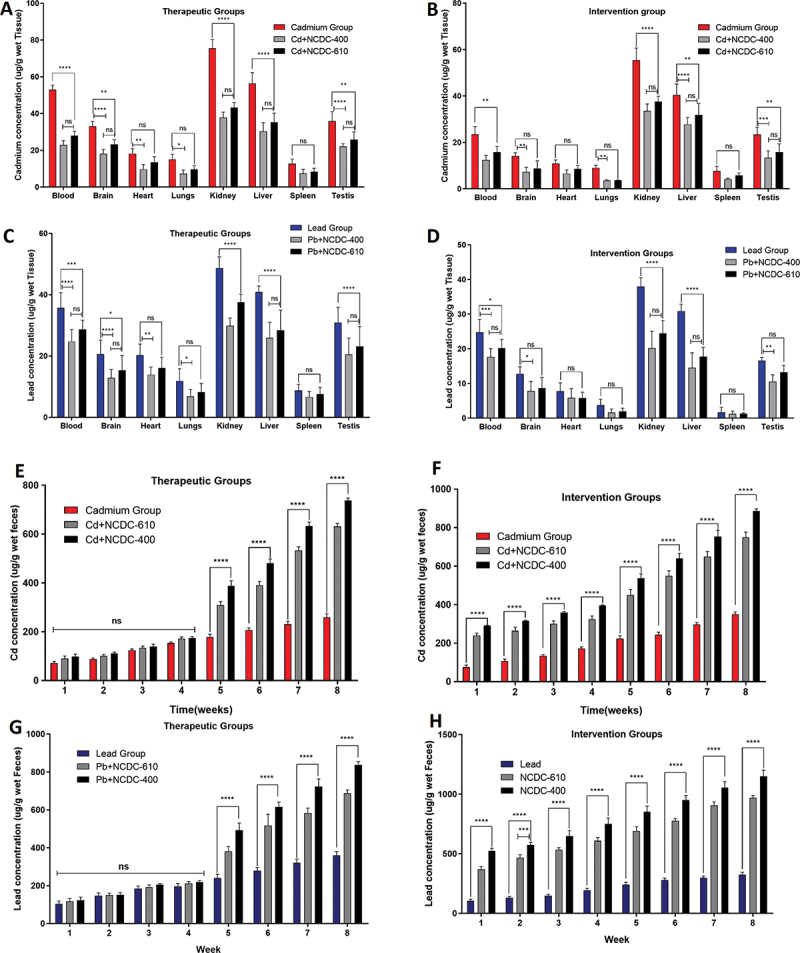
Effect of *L. Fermentum* (NCDC-400) *and L. rhamnosus* (NCDC-610) strains on the bioaccumulation of Cd and Pb in different organ systems of Wistar rats: (A) cadmium therapeutic groups, (B) cadmium intervention groups, (C) lead therapeutic groups, and (D) lead intervention groups. Effect of *L. Fermentum* (NCDC-400) *and L. rhamnosus* (NCDC-610) strains on Cd and Pb levels in the feces of Wistar rats. Cd and Pb levels were estimated in the feces of rats during the 8-week treatment in (E) cadmium therapeutic groups, (F) cadmium intervention groups (G) lead therapeutic groups, and (H) lead intervention groups. Data are shown as the mean±SEM values of the results from 8 rats per group. Asterisks indicate significant differences among groups (*p *< 0.05 *, *p *< 0.01 **, *p *< 0.001 ***, *p *< 0.0001 ****).

In the therapeutic group, both cultures demonstrated equal proficiency in substantially reducing the concentration of Cd in the bloodstream, as well as within the kidneys and liver. Similar results were observed in the intervention group, where both probiotic cultures performed equally well in reducing the bioaccumulation of Cd in the bloodstream, kidneys and liver. NCDC-400 performed significantly better than NCDC-610 in countering Cd accumulation in the brain, heart, lungs and testes in therapeutic group rats. Likewise, NCDC-400 significantly improved the Cd accumulation profile in the brain, lungs, liver and testes. No significant reduction in Cd accumulation was found in the spleen for either the therapeutic or intervention groups. The heart also showed a noteworthy reduction in the Cd profile by the use of probiotic culture in the intervention group (Figure 2A,B).

#### Lead

Similar to cadmium, the majority of lead (Pb) accumulation was observed in vital organs, namely, the bloodstream, kidneys, liver, and testes. In a therapeutic model, the utilization of cultures NCDC-400 and NCDC-610 yielded notable reductions in Pb accumulation profiles within the bloodstream, brain, kidneys, liver, and testes. Notably, there were no statistically significant differences in Pb levels observed in the brain, liver, testes, and bloodstream between NCD-400 and NCDC-610 cultures. In organs such as the heart and lungs, only cultured NCDC-400 was able to significantly reduce Pb compared to the control group. In an intervention model, organs including the heart, lungs, and spleen exhibited no significant differences among the experimental groups. However, both cultures demonstrated statistically significant differences in Pb levels within the bloodstream, kidneys, and liver. Remarkably, only the NCDC-400 culture achieved a significant reduction in Pb levels within the brain and testes (Figure 2E,F).

In summary, the data suggested a significant reduction in the accumulation of Cd and Pb in different organ systems of the treated rats. The Cd and Pb concentrations in the feces continued to decrease significantly during the first week after acute Cd exposure and gradually decreased over the 8 weeks in the heavy metal groups only (Figure 2C–H). The Cd and Pb concentrations in the feces were significantly higher in the groups treated with viable *L. fermentum* (NCDC-400) and *L. rhamnosus* (NCDC-610) strains.

**Figure 3.**
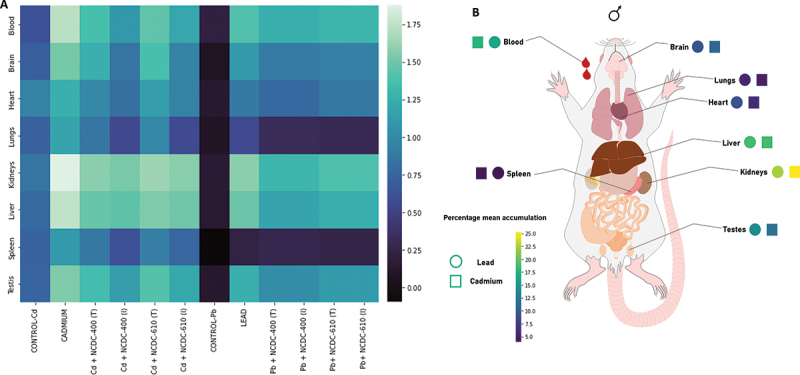
Bioaccumulation profile of Cd and Pb: (A) heatmap showing the accumulation of Cd and Pb in different Wistar rat groups. Log-transformed mean values from Cd and Pb estimation from different organs were used for heatmap construction. (B) Percentage mean accumulation of Cd and Pb (ppm) in different organ systems of Wistar rats.

Tracking the accumulation of Cd and Pb in different organs using atomic absorption spectroscopy, we found the highest accumulation of Cd and Pb in the kidney and liver. Figure 3A,B shows that the percentage accumulation of Cd and Pb is represented by a gradient color palette. The color palette for the data was generated after the analysis of the data in Python using the “Viridis” color palette. The percentage accumulation of Cd and Pb in decreasing order is as follows: kidney > liver > blood > testes > brain > heart > lungs > spleen.

### Measurement of oxidative stress using biochemical markers

#### Liver enzyme activities

**Figure 4.**
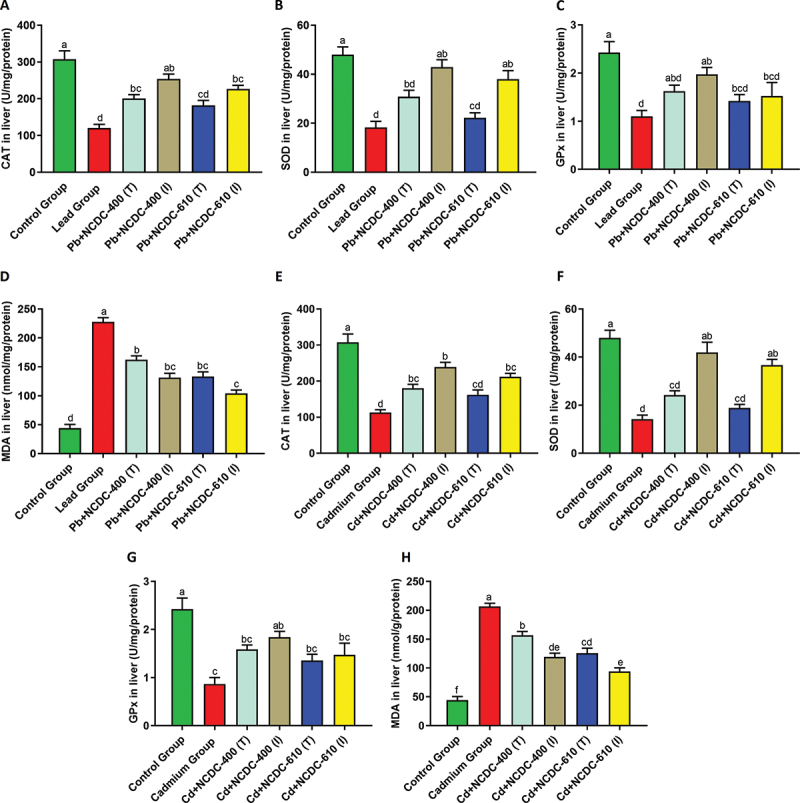
Antioxidant enzyme activity and MDA estimation in liver tissue of different Wistar rat groups: (A) catalase activity in Pb-treated groups (B) SOD activity in Pb-treated groups (C) GPx activity in Pb-treated groups (D) MDA levels in Pb-treated groups (E) catalase activity in Cd-treated groups (F) SOD activity in Cd-treated groups (G) GPx activity in Cd-treated groups (H) MDA levels in Cd-treated groups. Different letters on the bars indicate significant differences among groups with a *p* value <0.05.

Significant improvements in catalase activity were observed in both the NCDC-400 and NCDC-610 groups compared to the groups subjected to only Pb and Cd treatments. Interestingly, there were no significant differences among the NCDC-400 and NCDC-610 groups, regardless of whether they were part of the therapeutic or intervention models. This trend was observed in both Pb- and Cd-treated studies. The NCDC-610 (T) groups in the respective Cd and Pb study groups did not show notable improvement in enzyme activity. However, among the Pb- and Cd-treated mouse groups, NCDC-400 (I) showed significant recovery in enzyme activity. Additionally, the enzyme activity of Pb-NCDC-400 (I) was on par with that of the control that received no Pb treatment (Figure 4A,E).

Within the therapeutic model, neither the NCDC-400 (T) nor the NCDC-610 (T) groups demonstrated any significant improvements in SOD enzyme activity when compared to the Pb and Cd-treated groups. However, in the intervention model, both the NCDC-400 (I) and NCDC-610 (I) groups exhibited significant improvements in enzyme activity, which were comparable to the control group that did not receive any heavy metal treatment (Figure 4B,F). For the glutathione peroxidase enzyme, significant improvements in enzyme activity were observed only in the NCDC-400 (I) group. Other groups, including NCDC-400 (T), NCDC-400 (I), and NCDC-610 (T), did not display significant changes in enzyme activity (Figure 4C,G). In the case of malondialdehyde levels, significant reductions were evident in the NCDC-400 (T), NCDC-400 (I), and NCDC-610 (T) groups when compared to the Pb- and Cd-treated groups. However, it is important to note that malondialdehyde levels exhibited a particularly remarkable reduction in the NCDC-610 (I) group, surpassing the reductions observed in the other mentioned groups (Figure 4D,H).

### Kidney enzyme activities

**Figure 5.**
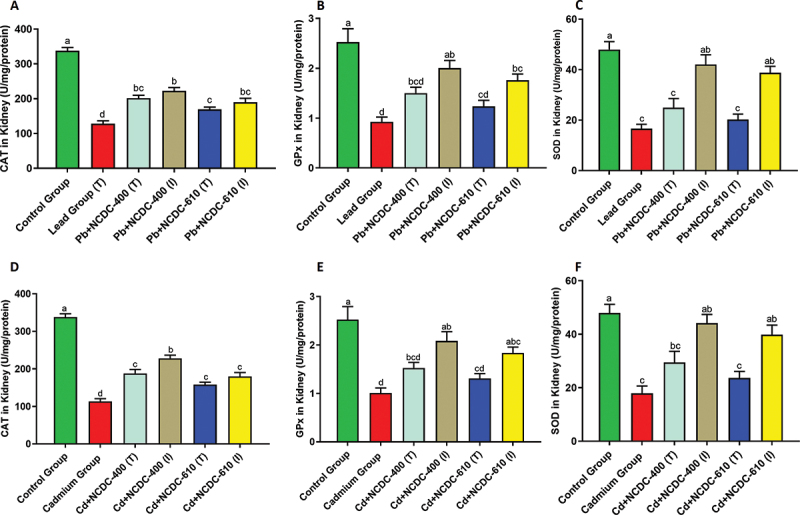
Antioxidant enzyme activity in kidney tissue of different Wistar rat groups: (A) catalase activity in Pb-treated groups (B) GPx activity in Pb-treated groups (C) SOD activity in Pb-treated groups (D) catalase activity in Cd-treated groups (E) GPx activity in Cd-treated groups (F) SOD activity in Cd-treated groups. Different letters on the bars indicate significant differences among groups with a *p* value <0.05.

The catalase enzyme activity showed significant improvement in the NCDC-400 (I) group in both Cd- and Pb-treated studies. The other groups *viz*. NCDC-400 (T), NCDC-610 (T) and NCDC −610 (I) exhibited significant improvement when compared to the heavy metal-treated groups, but they did not demonstrate any significant differences among themselves (Figure 5A,D). The Gpx showed significant improvement in activity in both the NCDC-400 (I) and NCDC-610 (I) groups compared to the heavy metal-treated (both Pb and Cd) groups. The NCDC-400 (T) and NCDC-600 (T) groups showed no significant improvement in either the Pb- or Cd-treated groups (Figure 5B,E). Similar to Gpx, a similar trend was observed for SOD in the Pb and Cd study groups, where NCDC-400 (I) and NCDC-610 (I) showed significant improvement compared to their Pb- and Cd-treated rat groups. Similar to previous observations with other enzymes, the NCDC-400 (T) and NCDC-610 (T) rat groups failed to show any significant improvement in enzyme activity.

### Histopathology of the liver and kidney

**Figure 6.**
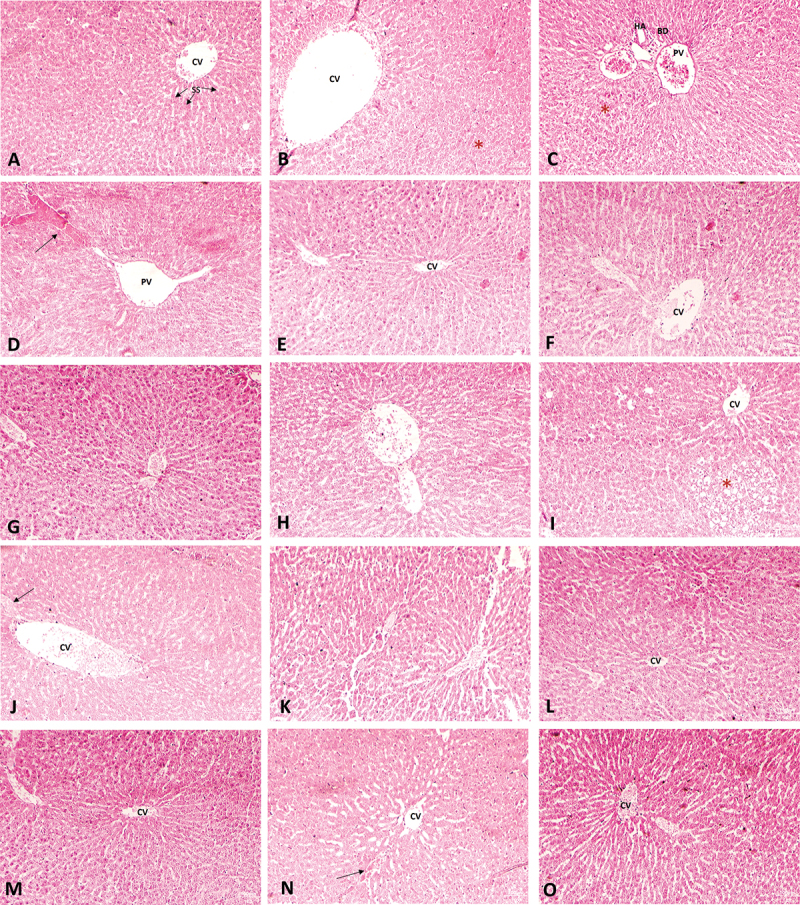
Representative images of A) histology of control rat group liver tissue; (B-D) liver tissue of the lead-treated rat group showing an extremely dilated central vein, bile duct proliferation, and congested portal space; therapeutic group: (E) NCDC-400 (F) NCDC-610; intervention group: (G) NCDC-400 (H) NCDC-610; (I-K) liver tissue of the cadmium-treated rat group showing necrotic hepatocytes, dilated and congested central veins and dilated sinusoidal spaces; therapeutic group: (L) NCDC-410 (M) NCDC-610; intervention group: (N) NCDC-410 (O) NCDC-610. CV: central vein; PV: portal vein; BD: bile duct; SS: sinusoidal spaces. The arrow marks the congested areas. The necrotic areas and areas with ballooning degeneration are denoted by (*).

Some major histological changes were observed during microscopic examination of liver tissue sections of the Cd- and Pb-treated rat groups, while the control rat group showed normal histological features of liver tissue. Compared to the control group, the lead-treated rat group showed highly dilated portal and central vein spaces with thin or damaged walls. The portal and central vein spaces were also found to be herniated and congested. The bile ducts found around portal veins in the vicinity of the hepatic artery showed proliferation (Figure 6A–D). The rats in the Pb-NCDC-400 group in both the therapeutic and intervention study design showed significant improvement or rather a normal liver histology (Figure 6E,G). However, the rats in Pb-NCDC-610 still showed some structural aberrations, such as a thin-walled herniated central vein with a mildly dilated space. Interestingly, this histological aberration was common in both therapeutic and intervention study models (Figure 6F,H). The cadmium-treated rats showed increased sinusoidal space dilation, ballooning degeneration and increased necrosis of hepatocytes. The central vein was characterized by a highly dilated and congested central vein with necrotic cells. A marked increase in herniated central and portal spaces was also evident in this group (Figure 6I–K). The Cd-NCDC-400 group (therapeutic) showed an improved central vein feature, but the sinusoidal spaces around the central vein were dilated with congestion in some areas (Figure 6L). In the case of the intervention study, a very small area showed sinusoidal dilation with necrotic cells, and other histological features appeared normal, which was a significant improvement over the cadmium-treated counterpart (Figure 6N). Cd-NCDC-610 improved the therapeutic model by restoring almost normal histology; however, in the intervention model, dilated sinusoidal spaces were still prevalent. Nonetheless, the Cd-NCDC-610 intervention model still showed relatively improved histological features when compared to cadmium-treated rat livers (Figure 6M,O).

**Figure 7.**
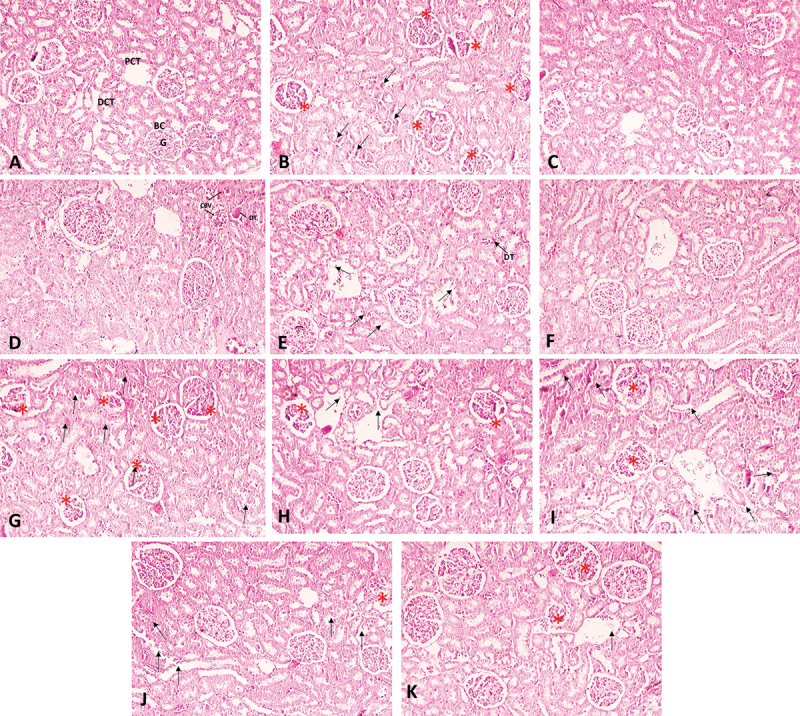
Representative images of A) histology of control rat kidney tissue; (B) kidney tissue of the lead-treated rat group showing severe atrophy of glomerular tufts with shrinkage and fragmentation (*), and desquamation and necrosis of tubular epithelial cells (arrow) therapeutic group: (C) NCDC-410 (D) NCDC-600; intervention group (E) NCDC-410 (F) NCDC-600; (G) kidney tissue of cadmium-treated rat group showing severe atrophy and degeneration of glomerular tufts with shrinkage, fragmentation and increased Bowman’s space (*), and desquamation and necrosis of tubular epithelial cells (arrow) Therapeutic; therapeutic group: (H) NCDC-410 (I) NCDC-600; intervention group (J) NCDC-410 (K) NCDC-600.

PCT: proximal convoluted tubule; DCT: distal convoluted tubule; G: glomerulus; BC: Bowman’s capsule; CBV: congested blood vessel, DT: degenerating tubule.

In the case of the kidneys, the lead-treated group showed a large number of glomeruli with shrinkage and fragmentation, and necrotic or sloughed-off epithelial cells were observed in the tubular lumen compared to the histology of the control group kidneys (Figure 7A,B).

In the Pb-treated groups, the probiotic treatment showed restoration of normal kidney histology with few minor histological aberrations, such as clogged blood vessels, degenerative tubules and necrosis of tubular epithelial cells; however, these changes were very scarce. A significant improvement in glomerular features was observed in the Pb-NCDC-400 and Pb-NCDC-610 groups in both the therapeutic and intervention study designs (Figure 7C–F). Similar to the Pb-treated group, the Cd-treated group showed a large number of completely atrophied glomerular tufts with desquamation and necrosis of tubular epithelial cells (Figure G). The NCDC-400- and NCDC-610-treated groups still showed degenerative or atrophied glomerular tufts; however, the number of such glomeruli was very low. In some areas, the tubular structure was also compromised with necrotic epithelial cells. A very similar histological pattern was observed in both prophylactic and therapeutic study models of the probiotic-treated groups. The probiotic treatment showed significant improvement in histological parameters; however, it was unable to completely restore the normal liver histology (Figure 7H–K).

## Discussions

Our study showed the abundant accumulation of heavy metals, i.e., cadmium and lead, in the liver and kidney, suggesting that these are the preferential sites for heavy metal accumulation. Several reports have suggested the predominant accumulation of Cd in the kidney and liver.^21,22^ Cd- and Pb-induced histomorphological changes in hepatic and renal tissues are discussed in a later section. Similar to our finding, one study assessing the distribution profile of Cd and Pb in different organs of Wistar rats showed higher accumulation in the liver and kidneys, while organs such as the brain, spleen and heart showed the least accumulation.^23^ The testes were next in line in the abundance of these heavy metals, followed by the brain, heart, lungs and spleen, in our investigation. Cd-induced toxicity has been shown to induce apoptosis in spermatozoa, reduce sperm mobility, and damage the blood‒testis barrier and seminiferous tubules, eventually resulting in the loss of germ cells.^14,15,24^ Similarly, chronic Pb exposure can result in a decrease in testosterone synthesis, degeneration of seminiferous tubules, reduced sperm motility and aberrant morphology.^25–27^ Therefore, chronic exposure to Cd and Pb can increase the risk of male infertility and thus can hinder the normal reproductive process. The spleen and lung showed relatively lower accumulation of heavy metals. As pointed out by the present study, the accumulation of heavy metals can be significantly reduced in different organs by competitive binding of probiotics with heavy metals and eliminating them through feces. In our study, significant amounts of Cd and Pb were excreted after 4 weeks in the therapeutic model and after one week in the interventional model.

**Figure 8.**
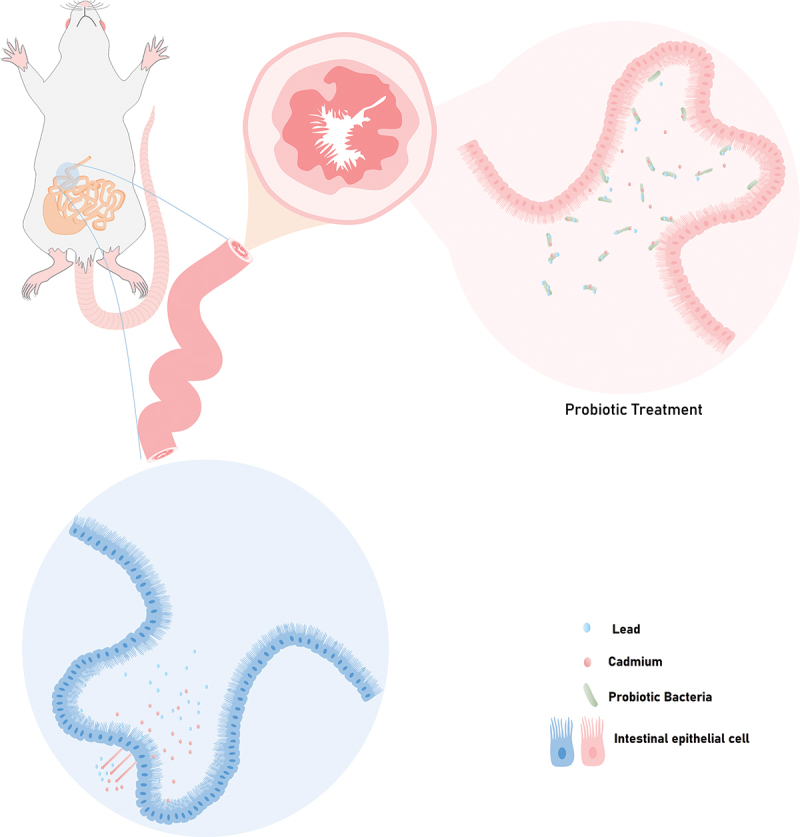
Probiotic strains check the accumulation of heavy metals by binding most of the heavy metals in the gut.

Due to their ability to chelate heavy metals, the gut microbiota can limit the absorption of heavy metals and thus check their level in blood circulation and different organs.^28^ In our study, the treatment of the two test probiotic cultures *viz*. NCDC-400 and NCDC-610 significantly reduced the accumulation of heavy metals in the organs by directing the heavy metals into the feces (Figure 8). Several studies have noted that this finding was in agreement with previous studies, where treatment with Lactobacillus as well as other probiotic strains significantly decreased the accumulation of heavy metals in different organs by directing a significant proportion of heavy metals through the feces.^22,29,30^ In a report, the accumulation of Cd in Cd-exposed mice was significantly lowered by the *L. plantarum* CCFM8610 strain.^31^ In another study, a combination of probiotics (*Lactobacillales, Clostridiales, Firmicutes*) was used to counter Cd toxicity by inhibiting Cd intestinal absorption.^32^

The histopathological data also indicated severe deterioration of these organs with evident structural changes in the tissue architecture. In rats treated with cadmium, there was notable damage to their liver, including sinusoidal space dilation, hepatocyte necrosis, and central vein congestion with necrotic cells. Additionally, this group exhibited a significant increase in herniated central and portal spaces. Improvement in histological features was observed in the Cd-NCDC-400 and Cd-NCDC-600 groups for both therapeutic and interventional models. However, the treatment of these cultures was not able to completely restore the normal histological features. Structural aberrations, such as dilated sinusoidal spaces lined with necrotic cells and mildly congested central vein spaces, were still observed. Similarly, the rats exposed to lead exhibited extensive dilation and damage to both portal and central vein spaces, with thin or weakened walls, as well as herniation and congestion in these areas. Additionally, proliferation of bile ducts near the portal veins in close proximity to the hepatic artery was observed. The probiotic treatment of Pb-treated rats significantly improved the histological features to an extent where they appeared normal. However, few mildly dilated portal and central vein spaces were observed in a few areas in the livers of rats treated with NCDC-400 and NCDC-610 in both therapeutic and intervention models. These changes were either mildly present or completely absent in the probiotic-treated rat group

Along with good heavy metal sequestration activity, the probiotic test culture also possessed good antioxidative properties. Further tissue damage by Cd and Pb can be caused by increased oxidative stress, which results in abnormal lipid peroxidation and decreased antioxidant enzyme activities. Heavy metals can significantly affect the antioxidative properties by inhibiting the sulfhydryl groups in enzymes viz. superoxide dismutase (SOD), glutathione peroxidase and catalase. The level of malondialdehyde, one of the final products of lipid peroxidation, was significantly reduced in mice administered NCDC-400 and NCDC-610 in the therapy and intervention groups. However, the NCDC-610 group in the intervention model showed a more significant reduction than the other groups for both Pb and Cd. The interventional treatment of NCDC-400 remarkably improved the enzyme activity for all the tested enzymes. The antioxidative capacities of various *Lactobacillus* strains have been reported, and these strains have been applied to alleviate heavy metal-induced oxidative stress.^22,33,34^ Studies have shown that probiotic treatment has a correctional effect on heavy metal-induced reduced activities of catalase, GPx, and SOD. These studies also reported reduced MDA levels after probiotic treatment, which is a marker of lipid peroxidation.^34,35^

*Lactobacillus* strains function as antioxidants by increasing the activities of antioxidant enzymes and inhibiting lipid peroxidation.^36^ By countering the increasing levels of Pb and Cd in organs with test culture, heavy metal-induced oxidative stress can be checked. Furthermore, it is still unclear whether such protection was simply a downstream effect of intestinal Cd and Pb sequestration or was due to direct protection related to the antioxidative abilities of NCDC-400 and NCDC-610 or both. One study reported that both intracellular cell free extract and intact cell of probiotic strains *Lactobacillus acidophilus* and *Bifidobacterium longum* possess the free radical scavenging activity. Both, cell free extract and intact cells were capable of inhibiting the peroxidation of linoleic acid.^37^ Additionally, certain strains of Lactobacilli have been demonstrated to elevate Nrf2 levels within the liver and kidneys of host. The Nrf2-Keap1-ARE system plays a crucial role in facilitating the synthesis of antioxidant enzymes within the cell. When reactive oxygen species (ROS) accumulate in the cell, the bond between Keap1 and Nrf2 is cleaved. Subsequently, the liberated Nrf2 is transported to the nucleus, where it binds to the antioxidant responsive element (ARE) and initiates the transcription of antioxidant enzymes encoded by ARE.^38,39^ Also, *lactobacilli* strains can produce their own antioxidant enzymes, for example *Lactococcus lactis* and *Lactobacillus fermentum* can produce CAT and Mn-SOD enzymes.^40,41^ This multifaceted mechanism may contribute to the observed improvement in antioxidant enzyme activity in the probiotic-treated group.

## Conclusions

The current study offers in-depth insights into the quantitative assessment of cadmium and lead levels within various organ systems. It also provides deleterious effects of their accumulation in two major organs viz. liver and kidney and how use of probiotic strains can limit the bioaccumulation of heavy metals. Both the NCDC-400 and NCDC-610 cultures successfully ameliorated the deleterious effects of Pb and Cd, but NCDC-400 performed relatively well when compared to NCDC-610. This might be due to their differential ability to sequester heavy metal ions. The regular use of probiotics can prevent the bioaccumulation of heavy metals and is a better way than the therapeutic strategy of eliminating heavy metal accumulation. Once accumulated, it is very difficult to completely remove or even deplete the systemic stores of heavy metals. Consistent probiotic treatment ensures the continuous removal of heavy metals from the gastrointestinal tract before they have the opportunity to be absorbed by the intestinal mucosa.
